# Identifying factors associated with COVID-19 related deaths during the first wave of the pandemic in Europe

**DOI:** 10.3389/fpubh.2022.922230

**Published:** 2022-07-28

**Authors:** Rainer Johannes Klement, Harald Walach

**Affiliations:** ^1^Department of Radiation Oncology, Leopoldina Hospital, Schweinfurt, Germany; ^2^Next Society Institute, Kazimieras Simonavicius University, Vilnius, Lithuania; ^3^Change Health Science Institute, Berlin, Germany

**Keywords:** Europe (Central), influenza vaccination, non-pharmaceutical interventions, SARS-CoV2, vitamin D

## Abstract

**Aim:**

To clarify the high variability in COVID-19-related deaths during the first wave of the pandemic, we conducted a modeling study using publicly available data.

**Materials and methods:**

We used 13 population- and country-specific variables to predict the number of population-standardized COVID-19-related deaths in 43 European countries using generalized linear models: the test-standardized number of SARS-CoV-2-cases, population density, life expectancy, severity of governmental responses, influenza-vaccination coverage in the elderly, vitamin D status, smoking and diabetes prevalence, cardiovascular disease death rate, number of hospital beds, gross domestic product, human development index and percentage of people older than 65 years.

**Results:**

We found that test-standardized number of SARS-CoV-2-cases and flu vaccination coverage in the elderly were the most important predictors, together with vitamin D status, gross domestic product, population density and government response severity explaining roughly two-thirds of the variation in COVID-19 related deaths. The latter variable was positively, but only weakly associated with the outcome, i.e., deaths were higher in countries with more severe government response. Higher flu vaccination coverage and low vitamin D status were associated with more COVID-19 related deaths. Most other predictors appeared to be negligible.

**Conclusion:**

Adequate vitamin D levels are important, while flu-vaccination in the elderly and stronger government response were putative aggravating factors of COVID-19 related deaths. These results may inform protection strategies against future infectious disease outbreaks.

## Introduction

The SARS-Corona-Virus 2 (CoV2) pandemic caused an unprecedented worldwide public health crisis by its impact on basically every system of human organization (1, 2). Untreated COVID-19 disease may lead to severe atypical pneumonia (3, 4), a cytokine storm and other potentially lethal sequelae (5–7). Other potential factors, such as host factors or population factors, were not much considered in the scientific and political discourse. For example, there is now strong evidence that vitamin D status predicts the risk for and outcome of COVID-19 infections (8–13). We also know that demographics play a role, as initially mostly elderly patients with a mean age above 70 years have been severely affected (14, 15). However, during the initial phase of the CoV2 outbreak, there was a wide variation in lethality across countries and regions. This variation is partially shrouded by the fact that most agencies and their dashboards propagate unstandardized figures of cases and deaths. A publication that estimated excess death rates in the US during the early time of the CoV2 pandemic as compared with the same months of previous years revealed a wide variation from−71,9 deaths per 100.000 inhabitants in North Dakota to 299,1 deaths per 100.000 inhabitants in New York City, with seven states actually exhibiting less excess mortality than in the previous comparison years, and 12 US states presenting with excess mortality figures below 10 per 100.000 inhabitants (16). The same is true for Europe: Miles and colleagues listed excess deaths of 21% for Spain, 20% for the UK, 18% for Italy down to 6% for Sweden, 3% for Portugal, −1% for Germany, −3% for Denmark and −4% for Norway during the first wave of the pandemic (17).

In order to be better prepared for future infectious disease outbreaks, there is clearly a need to understand what might have caused such variation in death numbers during the first wave of the pandemic. Are there population variables, public health variables, or individual-specific factors that can be identified that make this variation understandable? This was the guiding question of this modeling study.

## Materials and methods

We extracted data for 44 European countries for which the number of COVID-19 related deaths per 1.000.000 inhabitants up until 31^st^ August 2020 was known. This date was chosen since it approximately marked the end of the first infection wave in Europe (18, 19). The following 13 variables were used as putative predictors of the dependent variable “standardized COVID-19 related deaths” which we subsequently refer to as “y” (Supplementary material 1): (i) the test-standardized number of cases (in %), calculated as the number of cases in a country divided by the number of tests in that country × 100; (ii) the influenza vaccination rate in the elderly; (iii) life expectancy (in years); (iv) the population density (people per km^2^); (v) mean Government Response Stringency Index (GRSI) that describes the number and severity of non-pharmaceutical interventions employed between 15th March and 15th August 2020; (vi) vitamin D status (25(OH)D < 50 nmol/l vs. ≥ 50 nmol/l); (vii) cardiovascular disease (CVD) death rate; (viii) diabetes prevalence; (ix) smoking habits (average percentage of male and female smokers); (x) percentage of elderly (people older than 65 years); (xi) gross domestic product (GDP); (xii) human development index; (xiii) hospital beds (number of beds per thousand inhabitants). The data sources are given in Supplementary material 1.

**Figure 1:**
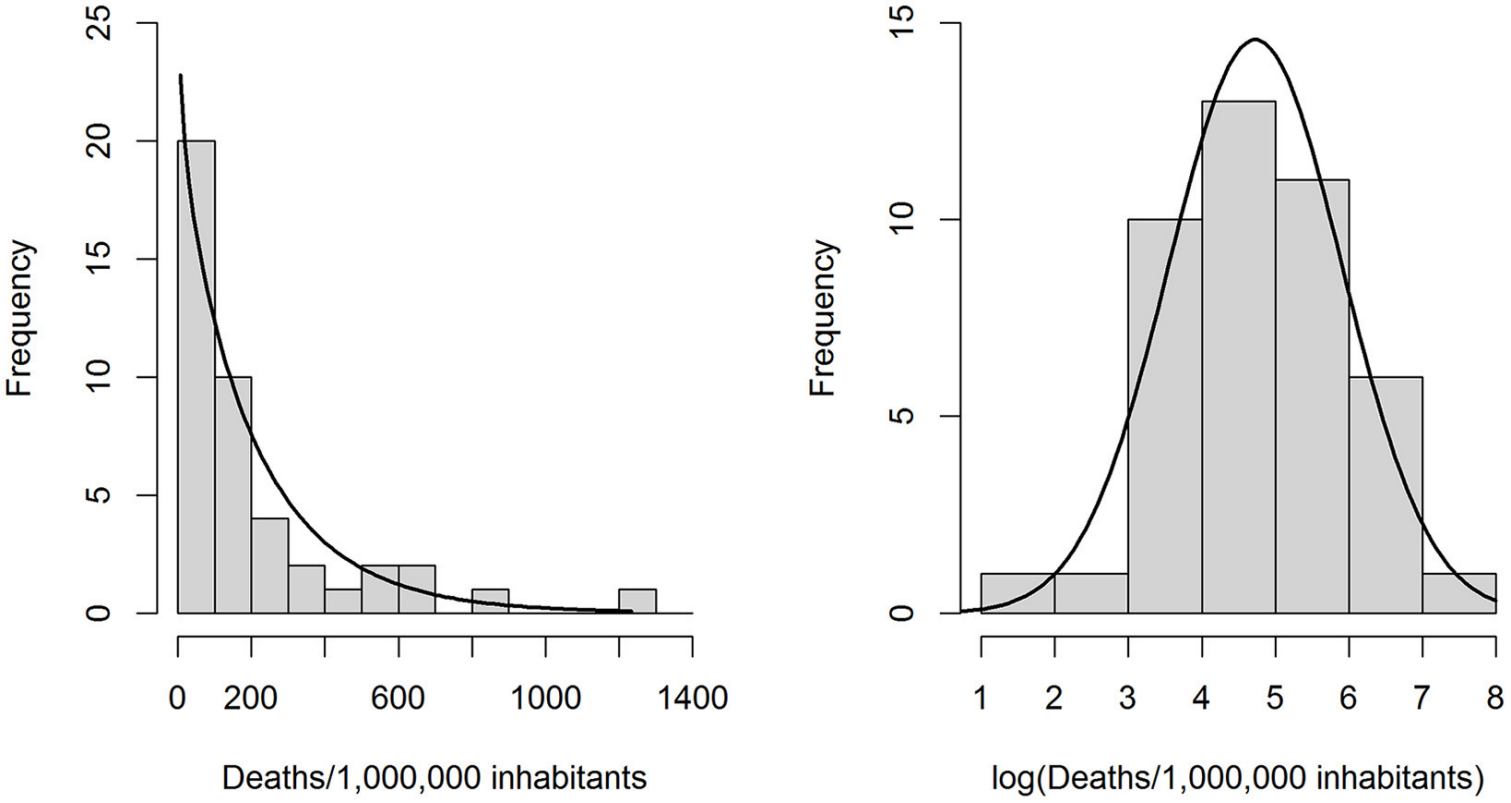
**Left:** Observed distribution of the outcome variable “COVID-19 related deaths per 1 million inhabitants” and a gamma distribution (rate = 0.00438, shape = 0.9270) fitted through maximum likelihood estimation. **Right:** Observed distribution of the log-transformed outcome variable, with a best-fit normal distribution.

Because the distribution of y followed a gamma distribution well (Figure 1), we calculated generalized linear models (GLMs) on a gamma-distributed variable with a log-link function. Since a log-transformation produced an outcome variable with an approximately normal distribution (Shapiro-Wilk normality test *p* = 0.864, Figure 1), we also calculated standard multiple linear regression models (LRMs) on log(y). During the initial check of modeling assumptions, one outlier (Andorra) was identified and removed from the sample (Supplementary material 1).

The final sample thus included 43 European countries of which 40 had known flu vaccination rates, 37 had known flu vaccination rates and GRSI values, and 31 had no missing variable values. To utilize as many cases as possible for multivariable modeling (20) missing variable values were imputed with multiple imputation by chained equations using the R package “mice” (21). A total of 100 imputation data sets were created. Each was used to fit the regression models, and the model parameters were averaged over all 100 model fits.

Different regression models were pre-specified according to plausible scientific hypothetical explanations for COVID-19 related deaths, reflecting the scientific practice of evaluating multiple pre-specified working hypotheses for their ability to explain the observed data (22). To complement the set of pre-specified hypotheses, one additional model was built using the Least Absolute Shrinkage and Selection Operator (LASSO), a variable selection method that shrinks the regression coefficients of less important predictors to zero (23). Rather than performing multiple null hypothesis testing, we ranked the different models according to their evidence by using the bias-corrected Akaike Information criterion (AICc) (22).

Because of the skewed distribution of some of the predictors, we first fitted a univariable model for each predictor and its log transform, and decided to use the latter for multivariable modeling if it resulted in a AICc reduction by at least 2 compared to its non-transformed values. In this way, it was found that test-standardized cases, population density and CVD death rate resulted in significantly better model fits when used as log-transformed variables.

As the simplest hypothesis, we assumed that the number of deaths could be predicted by the number of test-standardized cases:


(1) y∼log(test-standardized cases)

The second most-plausible simple hypothesis was that in addition to the number of cases, the severity of governmental responses, whose concept was to prevent infections, would allow better predictions of the outcome:


(2) y∼log(test-standardized cases)+GRSI

A third model was motivated by an interesting paper showing that the influenza vaccination rate in the elderly was significantly correlated at r = 0.68 with COVID-19 related deaths in Europe (24). Furthermore, early clinical data have indicated that vitamin D has protective effects against COVID-19, which would be expected based on its immune-modulatory functions (1). Finally, a modeling study by Liang et al. found that the number of hospital beds in a country was associated with decreased COVID-19 mortality (25). In model 3, we therefore investigated the impact of flu vaccinates rates, nation-wide vitamin D status and the number of hospital beds, which all could be seen as features of the health care system:


(3) $$\begin{array}{l} \text{y}∼\text{log}(\text{test}-\text{standardized cases})+\text{vitamin D status}\\ \text{ + hospital beds}+\text{flu vaccination rate} \end{array}$$

During the first wave of the pandemic, elderly people were far more susceptible to COVID-19 related deaths (19, 26), whereby an inverse association between vitamin D levels and COVID-19 severity was shown in an Italian study (27). These findings motivated the construction of a fourth model which attempted to predict COVID-19 related deaths based on vitamin D status and demographics:


(4) $$\begin{array}{l} \text{y}∼\text{log}(\text{test}-\text{standardized cases})+\text{vitamin D status}\\ \;+\text{life expectancy}+\text{percentage of elderly} \end{array}$$

Besides old age, it was soon revealed during the early phase of the pandemic that cardiovascular disease (19), diabetes (28) and smoking (29, 30) are associated with COVID-19 severity. The fifth hypothesis therefore assumed that population-level morbidity predictors would be relevant for predicting COVID-19 related deaths:


(5) $$\begin{array}{l} \text{y}∼\text{log}(\text{test}-\text{standardized cases})+\text{vitamin D status}\\ \;+\text{smoking habits}+\text{log}(\text{CVD death rate})\\ \;+\text{diabetes prevalence} \end{array}$$

Previous modeling studies also tested for associations between COVID-19 related deaths and different country-specific predictors such as the GDP and percentage of the elderly (25, 31). The sixth hypothesis therefore correlated COVID-19 deaths with such country-specific predictors:


(6) $$\begin{array}{l} \text{y}∼\text{log}(\text{test}-\text{standardized cases})+\text{vitamin D status}\\ \;+\text{log}(\text{population density})+\text{life expectancy}\\ \;+\text{GDP}+\text{human development index}\\ \;+\text{percentage of elderly} \end{array}$$

The seventh model was the full model using all 13 predictors which was included as a reference model (22).

Finally, an eighth model was built using a data-driven approach. To this aim, for each imputed dataset the most important variables were selected from the full set of 13 predictors using the LASSO method in a LRM predicting log(y). LASSO performs variable selection by shrinking the regression coefficients of the less important predictors to zero (32). The following variables were selected into the majority (>50) of models:


(7) $$\begin{array}{l} \text{y}∼\text{log}(\text{test}-\text{standardized cases})+\text{vitamin D status}\\ \;+\text{GRSI}+\text{flu vaccination rate}+\text{log}(\text{CVD death rate})\\ \;+\text{log}(\text{population density})+\text{GDP} \end{array}$$

The best model was identified as the one with the smallest AICc, and all other models were compared to the best model by computing AICc differences △_*i*_, probabilities *w*_*i*_ of model *i* being the best model (in the Kullback-Leibler information sense) and evidence ratios *E*_*i,j*_ = *w*_*i*_/*w*_*j*_ (22). Model adequacy was measured by R^2^, the proportion of variance explained by the predictors; for the GLMs a Kullback-Leibler divergence-based R^2^ measure was used (33).

All analyses were calculated with R version 4.0.2, and statistical significance was defined as *p*-values < 0.01. A detailed description of the statistics is given in Supplementary material 1.

## Results

**Figure 2:**
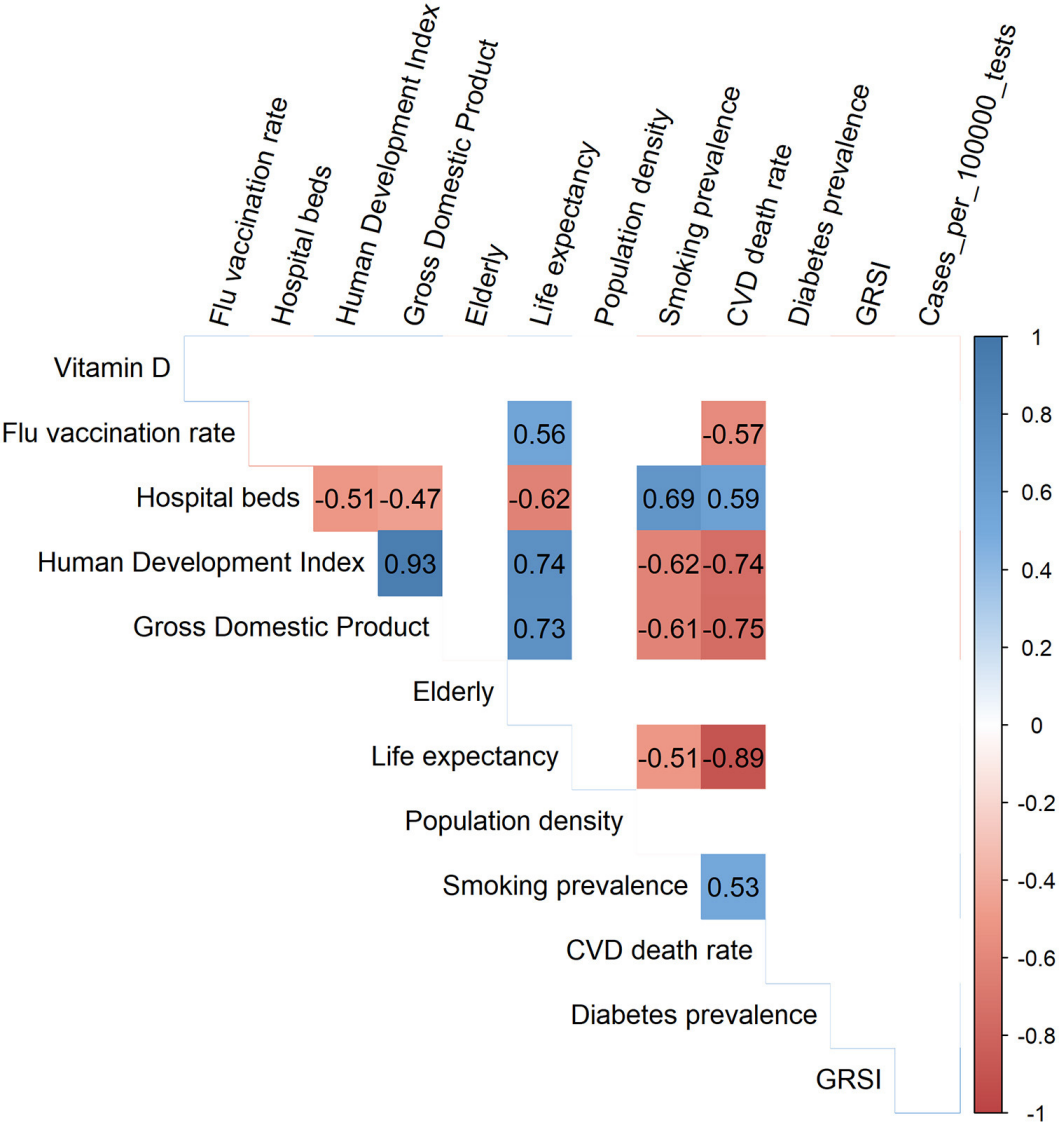
Corrgram showing the Spearman correlation coefficients for all significant correlations among the 13 variables used for modeling.

Figure 2 shows a so-called corrgram (34) for the 13 variables that we used for modeling, whereby only the significant (*p* < 0.01) correlations have been depicted. Smoking and CVD, but not diabetes prevalence, were inversely and significantly correlated with life expectancy, the human development index and gross domestic product. No significant correlations existed for vitamin D levels, test-standardized cases and the GRSIs with any of the other variables.

The results of both the GLMs (assuming a Gamma distribution for the outcome variable y) and the LRMs fitted to log(y) are presented in Table 1. The GLMs and LRMs yielded qualitatively similar results for all eight hypotheses considered. Test-standardized cases alone were able to explain about 20% of the variance in y or log(y), respectively, while the full model (model 7) was able to explain 64–67% as judged by the adjusted KL-R^2^ values. As expected, test-standardized cases were positively related to and the most significant predictor of COVID-19 related deaths in all models. As also expected, sufficient vitamin D status was associated with fewer deaths, although the association was only significant at the conventional p = 0.05 level in models 7 and 8, but not at *p* = 0.01, as stipulated. Surprisingly, however, it was found that the GRSI was no significant predictor of COVID-19 related deaths, and even exhibited a positive association (i.e., more stringent measures predicting higher death rates). Also surprisingly, flu vaccination rates were significantly and positively related to the outcome, i.e., there were more deaths in countries with higher flu-vaccination coverage. The number of hospital beds, percentage of elderly, human development index and smoking and diabetes prevalence, were not strongly associated with COVID-19 related deaths. In contrast, the GDP was found to be a significant predictor in models 7 and 8 with a higher GDP being associated with more COVID-19 related deaths.

**Table 1:** Parameters of the generalized linear models fitted to standardized deaths and linear models fitted to the logarithm of standardized deaths.

**Variable**	**Regression coefficient**	***p*** **-value**	**KL-R** ^2^	**KL-** ${\text{R}}_{\text{adj.}}^{2}$	**Regression coefficient**	***p*** **-value**	**R** ^2^	${\text{R}}_{\text{adj.}}^{2}$
Model 1	Generalized linear model				Linear model			
log(test-standardized cases [%])	0.600 (0.164)	0.00076	0.218	0.199	0.568 (0.165)	0.0014^*^	0.225	0.206
Model 2	Generalized linear model				Linear model			
log(test-standardized cases [%])	0.609 (0.159)	0.00047	0.297	0.261	0.498 (0.165)	0.0045^*^	0.286	0.250
GRSI	0.037 (0.017)	0.035			0.032 (0.017)	0.075		
Model 3	Generalized linear model				Linear model			
log(test-standardized cases [%])	0.729 (0.145)	1.9 × 10^−5^*^^	0.524	0.474	0.678 (0.157)	0.00013^*^	0.491	0.437
Vitamin D status (sufficient vs. deficient)	−0.299 (0.367)	0.425			−0.510 (0.348)	0.154		
Hospital beds per 1000	0.025 (0.068)	0.710			0.011 (0.064)	0.859		
Flu vaccination rate [%]	0.032 (0.007)	3.1 × 10^−5^*^^			0.029 (0.007)	0.00029^*^		
Model 4	Generalized linear model				Linear model			
log(test-standardized cases [%])	0.730 (0.131)	2.9 × 10^−6^*^^	0.498	0.446	0.695 (0.164)	0.00016^*^	0.424	0.363
Vitamin D status (sufficient vs. deficient)	−0.472 (0.287)	0.109			−0.416 (0.374)	0.275		
Life expectancy [years]	0.189 (0.040)	4.1 × 10^−5^*^^			0.155 (0.050)	0.0043^*^		
Elderly [%]	−0.033 (0.051)	0.533			0.005 (0.069)	0.943		
Model 5	Generalized linear model				Linear model			
log(test-standardized cases [%])	0.709 (0.151)	8.3 × 10^−5^*^^	0.551	0.490	0.735 (0.158)	5.3 × 10^−5^*^^	0.504	0.437
Vitamin D status (sufficient vs. deficient)	−0.363 (0.307)	0.248			−0.488 (0.346)	0.170		
Smoking prevalence [%]	−0.007 (0.029)	0.806			−0.006 (0.030)	0.844		
log(CVD death rate)	−0.999 (0.388)	0.020			−0.985 (0.392)	0.020		
Diabetes prevalence [%]	−0.09 (0.07)	0.185			−0.112 (0.080)	0.174		
Model 6								
log(test–standardized cases [%])	0.961 (0.171)	6.0 × 10^−6^*^^	0.557	0.484	0.894 (0.198)	0.00013^*^	0.516	0.436
log(population density [km^−2^])	0.222 (0.113)	0.058			0.127 (0.133)	0.325		
Life expectancy [years]	−0.011 (0.076)	0.883			0.011 (0.087)	0.886		
GDP	2.6 × 10^−5^ (1.5 × 10^−5^)	0.088			3.8 × 10^−5^ (1.7 × 10^−5^)	0.037		
Human development index	1.94 (5.52)	0.726			−3.9 (6.4)	0.549		
Elderly [%]	0.077 (0.062)	0.224			0.110 (0.074)	0.152		
Model 7								
log(test–standardized cases [%])	0.968 (0.148)	1.7 × 10^−6^*^^	0.773	0.671	0.951 (0.172)	1.3 × 10^−5^*^^	0.756	0.647
GRSI	0.030 (0.015)	0.055			0.030 (0.018)	0.118		
Vitamin D status (sufficient vs. deficient)	−0.716 (0.326)	0.043			−0.763 (0.372)	0.055		
Influenza vaccination rate	0.019 (0.006)	0.0055^*^			0.020 (0.007)	0.011		
Life expectancy [years]	−0.046 (0.112)	0.685			−0.070 (0.123)	0.575		
log(population density [km^−2^])	0.092 (0.107)	0.399			0.062 (0.124)	0.623		
Smoking prevalence [%]	−0.014 (0.022)	0.520			−0.022 (0.025)	0.350		
log(CVD death rate)	−0.037 (0.769)	0.962			−0.165 (0.936)	0.862		
Diabetes prevalence [%]	−0.058 (0.069)	0.414			−0.047 (0.082)	0.572		
Hospital beds per 1000	0.032 (0.072)	0.657			0.065 (0.082)	0.433		
GDP	3.8 × 10^−5^ (1.2 × 10^−5^)	0.0055^*^			4.2 × 10^−5^ (1.4 × 10^−5^)	0.0074^*^		
Human development index	−2.5 (5.2)	0.630			−2.8 (6.2)	0.654		
Elderly [%]	0.088 (0.057)	0.143			0.097 (0.065)	0.150		
Model 8								
log(test-standardized cases [%])	0.844 (0.123)	9.1 × 10^−7^*^^	0.727	0.681	0.809 (0.147)	1.1 × 10^−5^*^^	0.689	0.634
GRSI	0.021 (0.011)	0.071			0.021 (0.014)	0.154		
Vitamin D status (sufficient vs. deficient)	−0.704 (0.283)	0.023			−0.801 (0.332)	0.024		
Flu vaccination rate [%]	0.021 (0.005)	0.00021^*^			0.021 (0.006)	0.0015^*^		
log(population density [km^−2^])	0.127 (0.097)	0.209			0.099 (0.111)	0.379		
GDP	2.8 × 10^−5^ (7.3 × 10^−6^)	0.0070^*^			3.0 × 10^−5^ (9.0 × 10^−6^)	0.0023^*^		

*GDP, gross domestic product; GRSI, Government Response Stringency Index; CVD, Cardiovascular disease;*

* *p < 0.01 (statistically significant)*.

A ranking of the different models is given in Table 2. The evidence clearly favored the data-driven model 8 which was built by first selecting variables using the LASSO method. Compared with this model, all other models were basically ruled out by the strength of evidence. This shows that a specific combination of government-, population- and country-specific factors were important for predicting COVID-19-related deaths. This final model 8 was thereby able to explain roughly two-thirds of the variance in outcomes, similar to the full model 7, but using seven less predictors.

**Table 2:** Comparison of the eight different models specified in equations (1–8).

		**Generalized linear model**				**Linear regression model**				
**Rank**	**Model**	**AICc**	Δ_*i*_	*w*_*i*_	*E*_8,*i*_	**Model**	**AICc**	Δ_*i*_	*w*_*i*_	*E*_8,*i*_
1	8	505.1	0.0	0.9998	1	8	104.9	0	0.999	1
2	7	524.2	19.1	<0.0001	13,805	3	120.3	15.4	0.0005	2,211
3	3	524.8	19.7	<0.0001	19,376	7	121.8	16.9	0.0002	4,781
4	5	524.9	19.8	<0.0001	19,641	5	121.9	17.1	0.0002	5,083
5	6	527.5	22.42	<0.0001	73,783	6	123.9	19.1	<0.0001	13,940
6	4	527.5	22.43	<0.0001	74,275	4	125.7	20.8	<0.0001	33,247
7	2	538.5	33.4	<0.0001	>100,000	2	129.7	24.8	<0.0001	>100,000
8	1	541.3	36.2	<0.0001	>100,000	1	130.7	25.8	<0.0001	>100,000

*Models were ranked according to increasing AICc, i.e., the higher AICc the less parsimonious the model and the worse the fit in relation to the number of variables employed. AICc: Bias-corrected Akaike Information Citerion; △_i_: Difference in AICc to the best model (models with △ > 15–20 must be judged to be implausible); w_i_: probability of model i being the Kullback-Leibler best model; E_8,i_: evidence ratio between model 8 (the best model) and model i*.

In order to check if our results are dependent on the imputation of missing variables, we refitted the best GLM and LRM model to the original dataset with missing variables removed (Table 3). These models resulted in qualitatively very similar results as model 8 in Table 1 and confirmed that the two most important predictors of standardized COVID-19 related deaths were again influenza vaccination rates and the number of test-standardized cases, which were both positively associated with the outcome. GDP was also confirmed as a significant and positively associated predictor of COVID-19 deaths, and vitamin D status now reached the threshold of statistical significance in the GLM (*p* = 0.00573).

**Table 3:** Results of the full generalized linear models fitted to the original dataset with missing variables removed (intercept not reported).

	**Full generalized linear model (*****N** =* **31)**		**Full linear regression model (*****N** =* **31)**	
**Variables**	**Coefficient estimate (SE)**	***p*** **-value**	**Coefficient estimate (SE)**	***p*** **-value**
log(test-standardized cases [%])	0.812 (0.143)	7.6 × 10^−6^*^^	0.824 (0.174)	7.9 × 10^−5^*^^
GRSI	0.015 (0.011)	0.192	0.012 (0.014)	0.393
Flu vaccination rate [%]	0.026 (0.005)	8.6 × 10^−6^*^^	0.024 (0.006)	0.00026^*^
Vitamin D status (sufficient vs. deficient)	−0.777 (0.256)	0.0057^*^	−0.798 (0.311)	0.017
log (population density [km^−2^])	0.142 (0.097)	0.156	0.161 (0.117)	0.182
Gross domestic product	2.8 × 10^−5^ (7.3 × 10^−6^)	0.00064^*^	3.1 × 10^−5^ (8.8 × 10^−6^)	0.0016^*^
Model quality				
KL-R^2^	0.781		0.762	
Adjusted KL-R^2^	0.726		0.703	

*GRSI, Government Response Stringency Index; SE, Standard error;*

* *p < 0.01 (statistically significant)*.

## Discussion

Modeling COVID-19 related death rates in 43 European countries during the initial phase of the outbreak until August 2020, unravels some interesting findings:

a) Unsurprisingly, test-standardized CoV2-cases predict the number of deaths. This variable on its own was able to explain about 20% of the variance.
b) Surprisingly, the stringency of government responses correlated positively with COVID-19 related death rates, i.e., stricter government response was associated with more deaths; however, it was not a significant predictor.
c) Also surprisingly, the second-most important predictor was the flu-vaccination coverage in the elderly: the higher this vaccination rate is, the more COVID-19 related deaths we see in a country.
d) We confirmed that population-wide vitamin D status may have acted protectively against COVID-19 related deaths during the initial phase of the outbreak. It was a highly significant predictor in the best GLM fitted to the dataset with no missing variables (Table 3).
e) Countries with a higher GDP experienced a higher COVID-19 associated death rate.

These findings are strengthened by the fact that two different models reached the same conclusions: a GLM predicting a gamma-distributed outcome variable with log-linked predictors and a standard LRM with identity link functions of predictors on a log-transformed outcome variable.

It is easy to understand that more CoV2 cases translate into more COVID-19 related deaths. The importance of this predictor on its own is underlined by the fact that it is able to explain roughly 20% of COVID-19 related deaths. However, there remains variance to be explained by other factors. Although we do not assume we have captured all important variables, we have captured at least some as only six variables were able to explain about two-thirds of the total variance. A reassuring finding was that country-wide vitamin D status was inversely associated with COVID-19 related deaths, consistent with clinical and epidemiological data (8–13). Most surprising and most counterintuitive are the two findings that there are more COVID-19 related deaths in countries with higher flu vaccination coverage in the elderly, and, in addition, that the severity of governments' responses with non-pharmaceutical interventions was non-significant and counterintuitive in its effect (Table 1).

How can this strong association between flu vaccination rates and COVID-19 related deaths be explained? A careful randomized trial of flu vaccination in children showed that children who were vaccinated against influenza were better protected against influenza but suffered a fourfold higher risk of other respiratory virus dependent diseases (35). This might have to do with unknown mechanisms that disturb the ecology of pathogens, known as the virus interference phenomenon. A study conducted during the 2017/2018 influenza season revealed that flu vaccination was associated with a 36% increased odds of contracting respiratory coronavirus diseases (odds ratio 95% confidence interval 1.14–1.63, *p* < 0.01), while affording specific protection against influenza and parainfluenza viruses (36).

Thus, the negative impact of flu vaccination might have to do with several mechanisms: First, the virus interference phenomenon as shown for non-CoV2 coronaviruses (36); second, the fact that the immunological load on an organism that has to deal with a flu vaccine binds resources that cannot be mustered against a new and dangerous pathogen like CoV2. Third, it might also be the case that immune-enhancers in vaccines, such as aluminum derivates which are potentially toxic, burden the organism and hamper natural immunity. For example, it was shown experimentally in chicks that aluminum can disturb vitamin D metabolism (37). Furthermore, it has been argued that influenza vaccines are produced in eggs and other cell-systems that are not routinely tested against corona-viruses. Hence, corona-virus proteins from other corona-viruses might be present in these vaccines and induce allergic reactions against the novel CoV2 (38). Although these biological mechanisms would support the hypothesis that higher influenza vaccination rates increased COVID-19 mortality rates, we cannot rule out the possibility that influenza vaccination rate is simply a non-causal confounder strongly associated with some other (untested) variable, so that further research is needed to resolve this issue. Our finding is also in contrast to data from the US (39, 40). However, the correlation between influenza vaccination and COVID-19 death rate in the US is much lower than in Europe (24), probably because there is little variation in influenza vaccine coverage in the US. Our results are derived from population-level data in Europe in the elderly, which might describe a specifically susceptible fraction of the population.

Non-pharmaceutical interventions were widely hailed in modeling studies as having prevented higher incidence figures of cases and deaths (41–43). While this might be true for some countries and some single interventions, several authors are skeptical (44–49). Careful modeling studies for Germany, for instance, show that, although Germany was comparatively early to react – first measures were introduced on March 8 and shortly after this a full country lockdown was enacted – the peak of the infection and of the reproduction numbers was reached in nearly all 420 German districts on or around March 8 and thus none of the non-pharmaceutical interventions could have been causally related to the reduction of cases, and hence deaths (50, 51). The ensuing reduction of cases is a misattribution: it was not due to the lockdown, but obviously to the fact that the virus followed its own dynamic which needs to be better understood (52). Thus, there is independent evidence that non-pharmaceutical interventions are less effective than often thought. This would explain the weak association with COVID-19 related deaths in our analysis. Interestingly, our observation that the GRSI was positively associated with COVID-19 related deaths during the first phase of the pandemic replicates an earlier modeling study by Annaka which included data from 108 countries and in which this association was statistically significant in Ordinary Least Squares regression (31).

We find it quite remarkable that only six variables help to explain roughly two-thirds of the variation in COVID-19 related deaths. Because vitamin D status was one of them, it might be interesting to study other variables related to health. Vitamin D entered the best model number 8 with a comparatively large regression coefficient and was highly significant in a GLM fit to the complete dataset. Vitamin D seems to be an important predictor, as models without it are clearly inferior. For example, removing vitamin D status as a predictor from the GLM 8 fitted to the complete dataset resulted in a significantly worse model fit (AICc = 424.4 vs. 362.0) and less efficiency in explaining variance (KL-${\text{R}}_{\text{adj}}^{2}$ = 0.684 vs. KL-${\text{R}}_{\text{adj}}^{2}$ = 0.726). Thus, as a theoretically and numerically strong predictor, vitamin D strongly improves model fit and therefore we conclude that vitamin D was protective against death during the first wave of the Covid-19 pandemic. Its lack of strong statistical significance in models fit to the imputed dataset is likely due to the coarse grained nature of our data and uncertainty in imputation of missing values.

**Figure 3:**
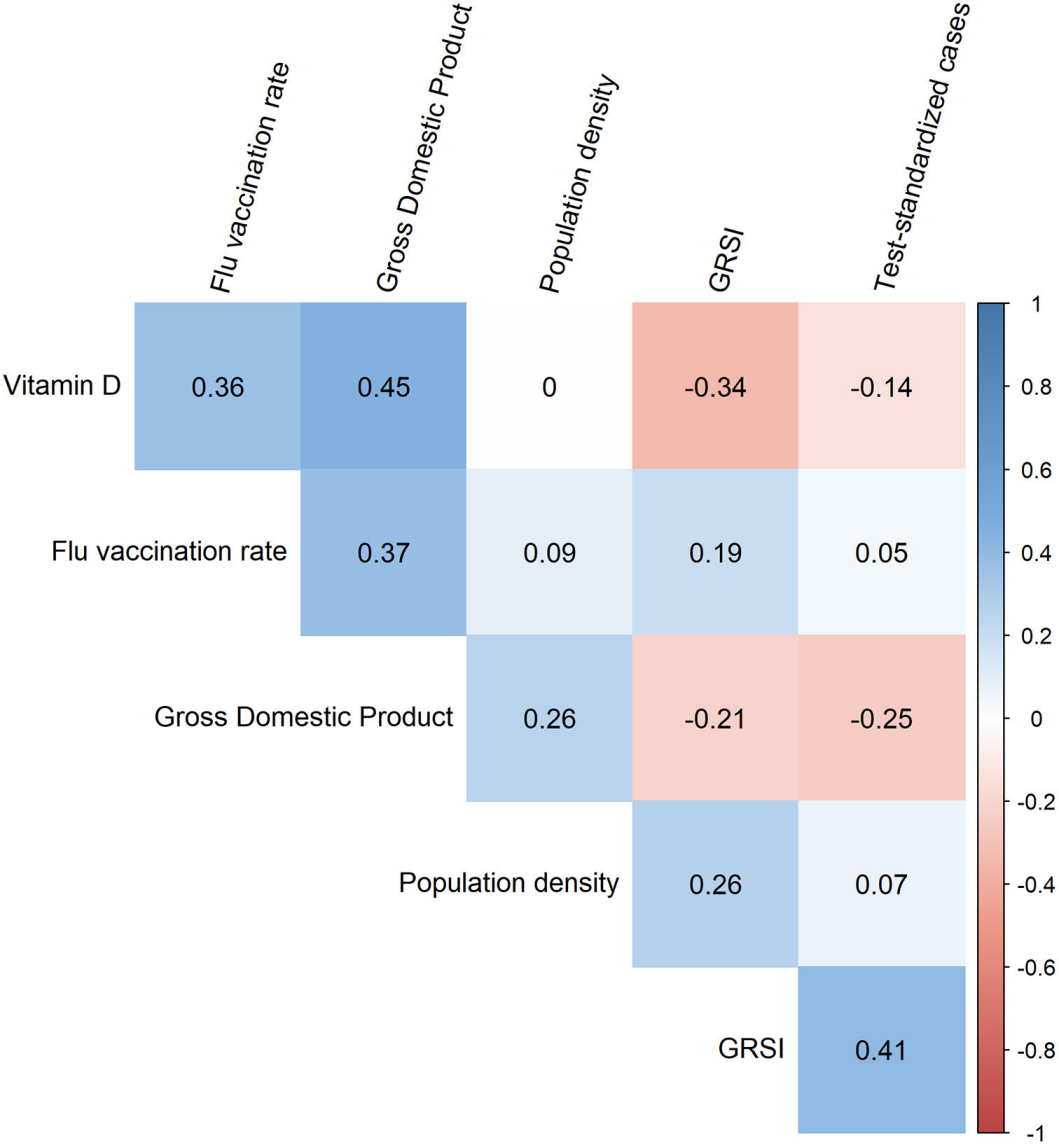
Corrgram showing the Spearman correlation coefficients for all pairs of the six variables included in the best model. The strongest correlation was observed between vitamin D and the gross domestic product which was almost significant (*p* = 0.013).

The limitations of our approach need to be kept in mind:

First, one might ask whether collinearity inflated our results, as some of the correlations between the variables used were rather high (Figure 2). However, this was not the case since no significant correlations existed among the six variables in the best model (Figure 3). This was supported by the computation of variance inflation factors which were all < 1.65, showing that there was no significant collinearity between these six variables. In particular, flu vaccination rate had the least collinearity with the other predictors (variance inflation factor = 1.2).

Second, we were unable to find flu vaccination data, GRSI and some other data for all countries. We tried to overcome this limitation through multiple imputation by chained equations, and the results were consistent with an analysis using only the cases for which every variable value was known.

Third, one potential problem we cannot remedy is the notorious unreliability of data or differences in the definition of cases, of deaths, and in reporting standards. This can be seen in the fact that Belgium is a clear outlier in all analyses that decreases the fit of the model. It is well known that the definition of COVID-19 related deaths in Belgium was more lenient than in other countries. Also, there is some evidence that some authoritarian governments tended to manipulate (downplay) their COVID-19 death data (31), which could have biased our results. In his country-level modeling study, Annaka (31) accounted for such a putative reporting bias by including the HRV transparency index developed by Hollyer et al. (53) which he used as a proxy for data transparency. However, the HRV index was originally not intended for assessing the transparency of pandemic death reporting; in addition, its latest version (the one used by Annaka) dates to the year 2010 and was only available for 21 of the 43 countries included in our analysis (median index 4.403, range −0.685–5.636); that Denmark scored worst with an HRV index of only −0.685 also appears counterintuitive and questions the application of this index to judge the transparency of COVID-19 related deaths reporting. The fact that we restricted the analysis to European countries of which the large majority nowadays is characterized as democratic would have mitigated the putative effects of data transparency bias.

Vitamin D estimates also have several uncertainties, such as having been measured in rather small cohorts, in different years and during different times of the year. Whenever possible, we preferred vitamin D values from the literature that had been measured in elderly people and during winter/spring. There was a weak positive correlation between a country's representative 25(OH)D concentration and latitude (Kendall's τ = 0.255, *p* = 0.0438), pointing toward vitamin D supplementation having a stronger influence on vitamin D status than living in southern latitudes. Also, COVID-19 reporting systems might be less reliable in some countries compared with others. These are the limits of our data and our analyses. But considering the fact that the whole world, politicians and public health officials use exactly the same data for their decisions should allow us to use them for analysis. One should remember that being a case, when considering the number of tests in a country, has only a weak relationship with becoming a fatality. It has been shown that the case fatality rate during the first wave was much less than previously assumed and estimated to be 0.15% (54). In Germany the case-fatality rate has been calculated from well documented cohorts to be 0.12 to 0.35% (55, 56). The still widely circulating higher case fatality rates are due to the fact that they are largely calculated using raw, absolute figures without knowledge of the real prevalence (57). But also standardized figures might be unreliable. Often the same person is tested multiple times. Thus, we likely overestimate the number of cases by some margin. This would mean: the true link between being a case and becoming a fatality is probably even weaker.

Considering all these weaknesses our paper also has some strengths. First, care was taken to ensure that the essential requirements for linear modeling were met. Second, we pre-specified plausible hypotheses (expressed as GLMs or LRMs) and used a robust model comparison framework based on Kullback-Leibler information to compare them, in this way automatically incorporating penalties for potential overfitting. Third, restricting the analysis to Europe means that we have a comparatively homogeneous sample which nevertheless has enough variability. While all countries issued warnings, the way non-pharmaceutical interventions were implemented differed widely, from suggestions and recommendations in Sweden to very strict stay-at-home orders that were policed in Spain, from nearly no regard in Belarus to strict political measures in Italy. Thus, we likely have seen a representative laboratory for the world, except that we do not cover any variance in ethnicity.

## Conclusions

In conclusion we see that COVID-19 related deaths during the first wave were most importantly dependent on the percentage of test-positive cases and flu-vaccination rate among the elderly in a country, whereby larger flu vaccination rates were associated with higher COVID-19 related deaths. The third important predictor was the GDP, followed by country-wide vitamin D status in the elderly, for which a causal relationship appears well supported by clinical and mechanistic evidence. These variables predict the variability in COVID-19 related deaths much better than the severity of governmental responses, the availability of hospital beds, smoking and diabetes prevalence or CVD death rates. Overall, we were able to show that a specific combination of government response-, population- and country-specific predictors was able to explain roughly two-thirds of the variance in COVID-19 related deaths. This might encourage others to look for additional factors that may explain the remainder of the variability in cases and deaths during the initial phases of the CoV2 outbreak. Ultimately, using the insights from modeling studies such as ours may help to be better prepared against future infectious disease outbreaks.

## Data availability statement

The original contributions presented in the study are included in the article/Supplementary material, further inquiries can be directed to the corresponding author/s.

## Author contributions

HW: conceptualization. RJK and HW: methodology, investigation, data curation, writing-original draft, and writing–review and editing. RJK: formal analysis. All authors contributed to the article and approved the submitted version.

## Conflict of interest

The authors declare that the research was conducted in the absence of any commercial or financial relationships that could be construed as a potential conflict of interest.

## Publisher's note

All claims expressed in this article are solely those of the authors and do not necessarily represent those of their affiliated organizations, or those of the publisher, the editors and the reviewers. Any product that may be evaluated in this article, or claim that may be made by its manufacturer, is not guaranteed or endorsed by the publisher.
