# Supplementary material for: Identifying factors associated with COVID-19 related deaths during the first wave of the pandemic in Europe
Source: Front Public Health. 2022 Jul 28;10:922230. doi: 10.3389/fpubh.2022.922230 (PMC9366394; doi:10.3389/fpubh.2022.922230)
Supplement: Supplementary file 1 [file Data_Sheet_1.docx]

# Supplementary Data File 1

## Data Sources

We retrieved data on standardized COVID-19 cases and deaths (per 1.000.000 inhabitants) as well as all other variables from an Excel spreadsheet downloaded from <https://ourworldindata.org/covid-vaccinations> (accessed on 1^st^ of April 2022) for 44 European countries^[[1]](#footnote-1)^. Since numbers of deaths were zero for Channel Islands, Faroe Islands and Gibraltar, these three European countries were not considered because the appropriate model, a generalized linear model to predict a gamma-distributed variable, does not allow for zeros in the predictor variable. A total of seven monthly values for the Government Response Stringency Index (GRSI) for each country (Hale et al. 2021), ranging between February and August 2020, were retrieved from the COVID-19 Government Response Tracker website at <https://www.bsg.ox.ac.uk/research/research-projects/covid-19-government-response-tracker> (accessed 15^th^ March 2021). The selected monthly GRSI values corresponded to the 15^th^ day of each month from February to August; these seven values were averaged to obtain an overall measure representing the stringency of policies within our timeframe of interest. No GRSI values were available for Isle of Man, Liechtenstein, Montenegro and North Macedonia.

An interesting paper showed that the influenza vaccination rate in the elderly was significantly correlated at r = .68 with Covid-19 related deaths in Europe (EBMPHET Consortium 2020). We therefore extracted flu vaccination rates in the elderly (usually in persons aged 65 and older) from the OECD website (<https://data.oecd.org/healthcare/influenza-vaccination-rates.htm>) (accessed on 1^st^ of April 2022). These rates referred to the year 2019 or the latest available. The latest available flu vaccination rate for Liechtenstein (2018) was retrieved from EUROSTAT (<https://ec.europa.eu/eurostat/de/web/products-eurostat-news/-/DDN-20200915-1>, accessed on 1^st^ of April 2022). We were not able to locate influenza vaccination data for Bosnia, Moldovia and Monaco.

Finally, latitudes for the capital city in each country were obtained from Panarese and Shahini (Panarese and Shahini 2020), and country-wide estimates of 25(OH)D concentrations in older adults were retrieved from various sources. If possible, 25(OH)D concentrations measured in adults >60 years during winter time were preferred as they would better reflect levels at the time of the SARS-CoV2 outbreak. For modelling, 25(OH)D concentrations were dichotomized into a vitamin D status indicator variable with values 0 (vitamin D deficient; 25(OH)D<50nmol/l) and 1 (vitamin D sufficient; 25(OH)D≥50nmol/l).

Supplementary Table S1 shows the distribution of all variables used as predictors of Covid-19 related deaths, and Supplementary Table S2 gives the most relevant data that we collected from the various sources.

## Statistics

The outcome of interest for this modeling study was the number of deaths per 1.000.000 inhabitants. Because the distribution of y followed a gamma distribution well (Figure S1), we calculated generalized linear models (GLMs) on a gamma-distributed variable with a log-link function. Since a log-transformation produced an outcome variable with an approximately normal distribution (Shapiro-Wilk normality test p=0.635, Figure S2), we also calculated standard multiple linear regression models (LRMs) on log(y). Prior to modelling, we checked that the basic assumptions for multiple regression models would hold: Linear relations of each predictor with the outcome were checked by creating scatter plots with a fitted linear regression line, and the assumption of a constant variance of the response variable around the regression line was checked by inspecting residual plots of a GLM and LRM fitted with log(test-standardized cases), log(population density), life expectancy and latitude as predictors. These predictors were chosen because they were known for all countries, and latitude was used as a proxy for vitamin D status, which we found could be predicted by the equation Pr(vitamin D sufficiency) = logit^-1^(−7.647+0.139×latitude[°]) with a p-value of 0.0231 for latitude. Residual plots revealed that one country (Andorra) was an outlier with large standardized residual close to 3 in both the GLM and LRM; it was therefore removed from the dataset.


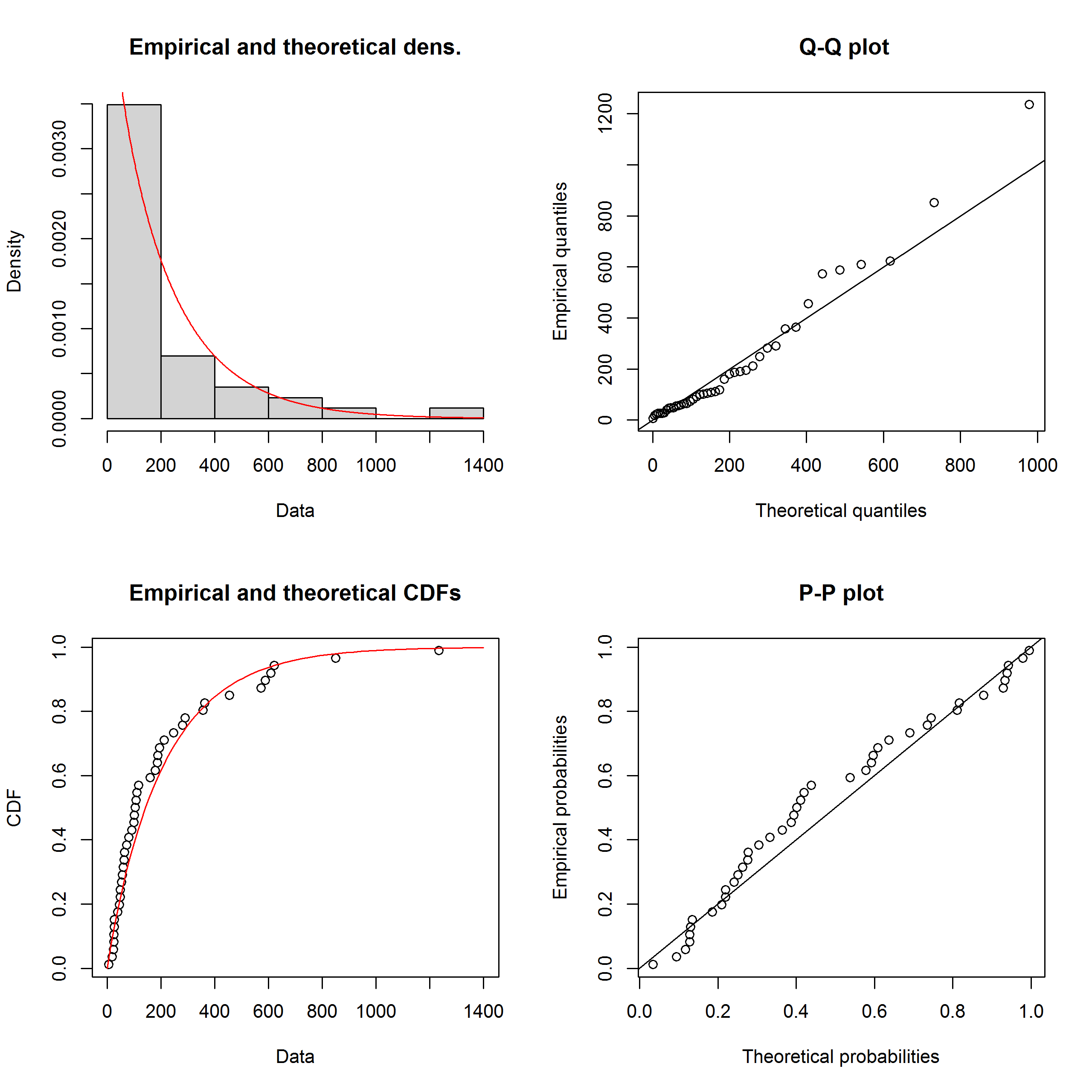


**Figure S1**: Fitting a gamma distribution to the response variable “Deaths per 1,000,000 inhabitants” by maximum likelihood estimation

The final sample thus included 43 countries of which 40 had known flu vaccination rates, 37 had known flu vaccination rates and GRSI values, and 31 had no missing covariate values. To utilize as many cases as possible for multivariable modeling (Sterne et al. 2009), missing covariates were imputed with multiple imputation by chained equations using the R package ‘mice’ (Van Buuren and Groothuis-Oudshoorn 2011). A “missing at random” mechanism was assumed being responsible for missing variables, with all putative predictor variables given in Supplementary Table S1 plus latitude and the outcome variable (deaths per 1.000.000 inhabitants) being added into the imputation model. Variables were imputed in the order of their number of missing cases. Predictive mean matching was used for imputing flu vaccination rates and GRSI and logistic regression for predicting vitamin D status, respectively. A total of 100 imputation data sets were created. Each was used to fit generalized linear regression models, and the model parameters were averaged over all 100 model fits.


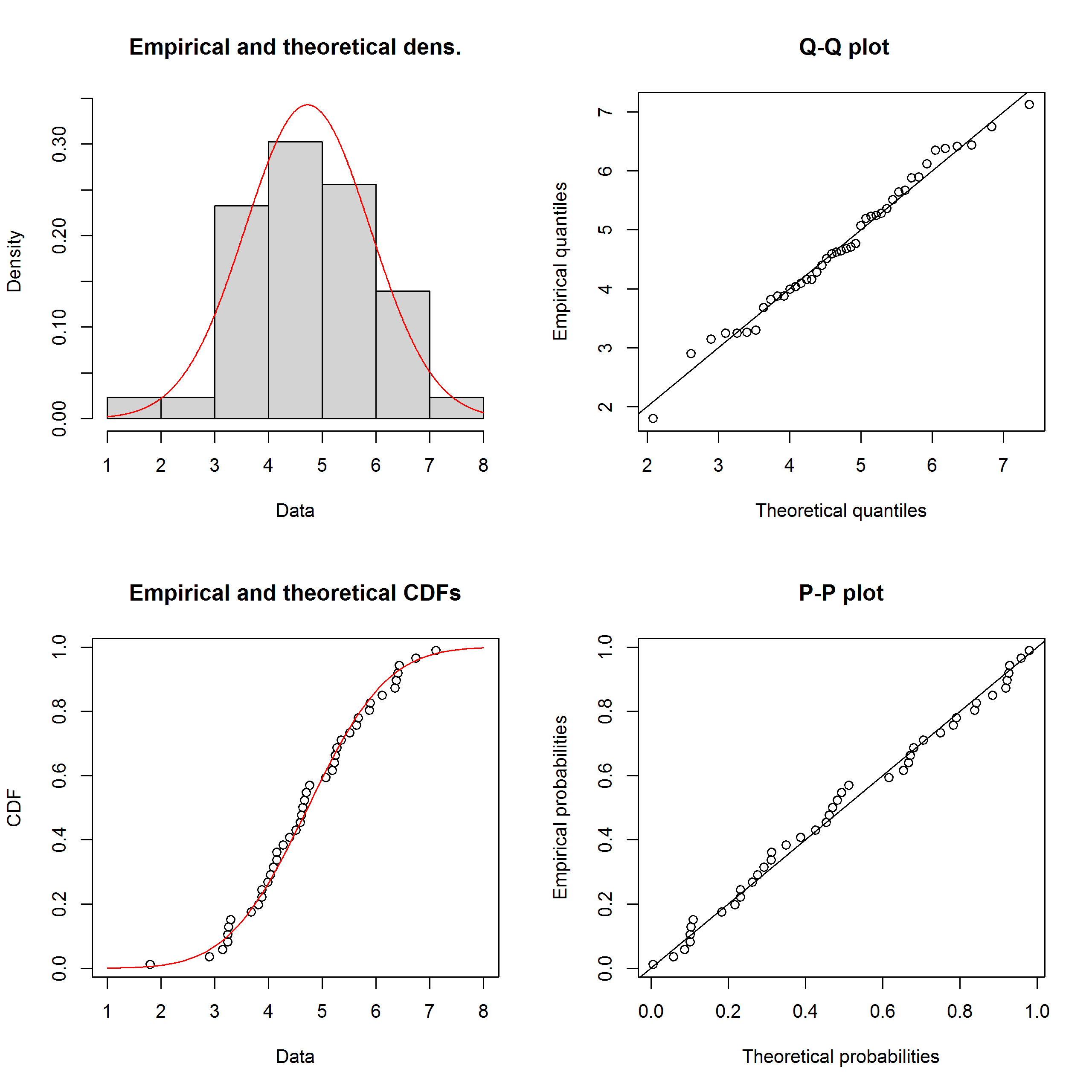


**Figure S2**: Fitting a normal distribution to the log-transformed response variable

Since our goal was to explain the variation in Covid-19 related deaths between European countries with the extracted variables, we posed several plausible model hypotheses and compared them with respect to their ability to predict the data using the bias-corrected Akaike Information criterion (Anderson 2008). As the simplest hypothesis, it was assumed that the number of standardized deaths could be predicted by the number of test-standardized cases:

y $\sim$ log(test-standardized cases) (1)

Seven other plausible hypotheses were subsequently formulated as described in the main paper.

The best model was identified as the one with the smallest AICc, and all other models were compared to the best model by computing AICc differences $\triangle_{i}$, probabilities $w_{i}$ of model $i$ being the best model (in the Kullback-Leibler information sense) and evidence ratios $E_{i,j}=\frac{w_{i}}{w_{j}}$ (Anderson 2008). Model adequacy was measured by R^2^ , the proportion of variance explained by the predictors; for the GLMs a Kullback-Leibler divergence-based R^2^ measure was used (Colin Cameron and Windmeijer 1997).

All analyses were calculated with R version 4.0.2, and statistical significance was defined as p-values <0.01.

**Supplementary Table S1**: Distribution of the variables used for predicting Covid-19 related death

| **Variable** | **N** | **Median** | **Mean** | **Range** | **log-transformation for modeling** |
| --- | --- | --- | --- | --- | --- |
| Test-standardized cases [%] | 43 | 3.1 | 4.4 | 0.25-17.5 | Yes |
| GRSI | 40 | 51.0 | 49.9 | 13.4-64.7 | No |
| Hospital beds | 42 | 4.52 | 5.21 | 2.22-13.8 | No |
| Flu vaccination rate | 40 | 34.1 | 37.3 | 0.1-75.5 | No |
| Vitamin D status  *Deficient*  *Sufficient* | 32  20  12 |  |  |  | No |
| Smoking prevalence [%] | 38 | 29.3 | 29.2 | 14.8-45.9 | No |
| Diabetes prevalence [%] | 42 | 5.79 | 6.46 | 3.28-10.08 | No |
| CVD death rate | 39 | 175.7 | 235.5 | 56.1-539.9 | Yes |
| Life expectancy [years] | 43 | 81.3 | 79.7 | 71.9-86.8 | No |
| Elderly (age >65) [%] | 39 | 18.6 | 17.8 | 10.9-23.0 | No |
| Population density [km^-2^] | 43 | 104.9 | 607.7 | 3.4-19348 | Yes |
| Gross domestic product [$] | 40 | 30778 | 33973 | 5190-94278 | No |
| Human Development Index | 40 | 0.89 | 0.88 | 0.75-0.957 | No |

CVD: Cardiovascular disease

**Supplementary Table S2**

| **Country** | **y** | **Vitamin D**  **[nmol/l]** | **Vitamin D source** | **GRSI** | **Influenza**  **Vaccination**  **rate [%]** | **Cases per Test [%]** | **Latitude [°]** | **Smoking**  **prevalence [%]** | **Population**  **density** |
| --- | --- | --- | --- | --- | --- | --- | --- | --- | --- |
| Albania | 98.854 | 47.5 | (Rumano et al. 2019) | 64.68 | 11.8 | 16.33 | 41 | 29.15 | 104.871 |
| Andorra* | 685.162 | NA | NA | 36.11 | 30.2 | 0.82 | 42 | 33.4 | 163.755 |
| Austria | 81.057 | 42 | (Elmadfa et al. 2018) | 43.26 | 20.3 | 2.32 | 47 | 29.65 | 106.749 |
| Belarus | 72.118 | 65 | (Lips et al. 2019) | 13.36 | 75.5 | 4.71 | 55 | 28.3 | 46.858 |
| Belgium | 850.646 | 49.3 | (Lips et al. 2019) | 54.10 | 59.1 | 3.74 | 50 | 28.25 | 375.564 |
| Bosnia and Herzegovina | 186.612 | 48.3 | (Sokolovic et al. 2017) | 55.49 | NA | 10.76 | 44 | 38.95 | 68.496 |
| Bulgaria | 91.204 | 38.75 | (Borissova et al. 2013) | 44.18 | 7.7 | 4.01 | 42 | 37.25 | 65.18 |
| Croatia | 45.57 | 46.9 | (Lips et al. 2019) | 50.93 | 34 | 6.07 | 45 | 37.1 | 73.726 |
| Czechia | 39.535 | 62.5 | (Lips et al. 2019) | 46.63 | 21.5 | 2.70 | 50 | 34.4 | 137.176 |
| Denmark | 107.34 | 47.8 | (Spiro and Buttriss 2014) | 51.85 | 52 | 0.70 | 56 | 19.05 | 136.52 |
| Estonia | 48.295 | 42 | (Lips et al. 2019) | 36.77 | 10.2 | 1.60 | 59 | 31.9 | 31.033 |
| Finland | 56.593 | 67.7 | (Lips et al. 2019) | 39.42 | 49.5 | 1.27 | 63 | 20.45 | 35.308 |
| France | 454.54 | 60 | (Lips et al. 2019) | 55.23 | 51 | 4.41 | 46 | 32.85 | 18.136 |
| Germany | 110.822 | 50.1 | (Rabenberg and Mensink 2016) | 50.99 | 34.8 | 2.17 | 51 | 30.65 | 122.578 |
| Greece | 25.649 | 47.3 | (Lips et al. 2019) | 51.85 | 56.2 | 1.07 | 39 | 43.65 | 237.016 |
| Hungary | 63.835 | 48.4 | (Lips et al. 2019) | 51.19 | 24.1 | 1.41 | 47 | 30.8 | 3457.1 |
| Iceland | 27.116 | 57 | (Lips et al. 2019) | 38.76 | 47.5 | 0.99 | 65 | 14.75 | 83.479 |
| Ireland | 356.619 | 56.4 | (Lips et al. 2019) | 58.00 | 68.5 | 3.48 | 53 | 24.35 | 108.043 |
| Isle of Man | 280.998 | NA |  | NA | 72 | 3.54 | 54 | NA | 3.404 |
| Italy | 587.783 | 37.9 | (Spiro and Buttriss 2014) | 60.85 | 53.1 | 3.14 | 42 | 23.8 | 69.874 |
| Latvia | 18.212 | NA |  | 47.88 | 11.7 | 0.56 | 57 | 38.3 | 147.872 |
| Liechtenstein | 26.141 | NA |  | NA | 20.2 | 11.89 | 47 | NA | 205.859 |
| Lithuania | 25.652 | 46.8 | (Bleizgys and Kurovskij 2018) | 43.12 | 14.8 | 0.45 | 55 | 29.65 | 31.212 |
| Luxembourg | 195.333 | 53.6 | (Alkerwi et al. 2015) | 42.46 | 39.8 | 1.05 | 50 | 23.45 | 237.012 |
| Malta | 23.251 | NA |  | 50.79 | 42.5 | 0.99 | 35 | 25.55 | 45.135 |
| Moldova | 247.265 | NA |  | 62.96 | NA | 17.51 | 47 | 25.25 | 231.447 |
| Monaco | 101.215 | NA |  | 64.42 | NA | 0.25 | 44 | NA | 1454.037 |
| Montenegro | 159.223 | NA |  | NA | 15.8 | 9.84 | 43 | 45.95 | 123.655 |
| Netherlands | 363.417 | 64.7 | (Lips et al. 2019) | 48.81 | 62.7 | 4.78 | 52 | 25.85 | 19347.5 |
| North Macedonia | 289.533 | NA |  | NA | 11.8 | 9.72 | 41 | NA | 46.28 |
| Norway | 48.302 | 65 | (Lips et al. 2019) | 46.16 | 38.2 | 1.55 | 64 | 20.15 | 508.544 |
| Poland | 53.946 | 32.5 | (Lips et al. 2019) | 51.45 | 6.87 | 2.51 | 52 | 28.2 | 82.6 |
| Portugal | 179.191 | 42.3 | (Santos et al. 2017) | 58.33 | 60.8 | 2.91 | 40 | 23.15 | 14.462 |
| Romania | 189.306 | NA |  | 52.25 | 23.5 | 4.83 | 46 | 30 | 124.027 |
| Russia | 117.386 | 25 | (Lips et al. 2019) | 59.72 | 36.2 | 2.72 | 65 | 40.85 | 112.371 |
| San Marino | 1234.931 | NA |  | 47.62 | 31.1 | 10.63 | 44 | NA | 85.129 |
| Serbia | 103.761 | NA |  | 52.64 | 7.8 | 3.40 | 44 | 38.95 | 8.823 |
| Slovakia | 6.056 | 81.5 | (Lips et al. 2019) | 46.96 | 12.5 | 1.16 | 49 | 30.4 | 556.667 |
| Slovenia | 63.982 | 47.7 | (Hribar et al. 2020) | 43.45 | 12.9 | 1.81 | 46 | 22.55 | 80.291 |
| Spain | 622.395 | 42.9 | (Spiro and Buttriss 2014) | 61.57 | 54.9 | 5.35 | 39 | 29.4 | 113.128 |
| Sweden | 572.924 | 68.7 | (Lips et al. 2019) | 47.88 | 52.2 | 7.67 | 59 | 18.85 | 102.619 |
| Switzerland | 212.036 | 46 | (Ilie et al. 2020) | 41.93 | 32 | 4.16 | 47 | 25.75 | 93.105 |
| UK | 609.159 | 47.4 | (Lips et al. 2019) | 53.84 | 72 | 2.04 | 55 | 22.35 | 24.718 |
| Ukraine | 59.931 | 26 | (Lips et al. 2019) | 56.48 | 0.07 | 7.75 | 49 | 30.45 | 214.243 |

In most cases, vitamin D levels are mean values of specific cohorts reported in the literature; in some cases, they represent median values. Variables not given here can be obtained from the MS Excel file on the Our World in Data webpage (<https://ourworldindata.org/covid-vaccinations>; accessed on 1^st^ of April 2022). *Andorra was an outlier and removed from the regression analysis

## References

Alkerwi A, Sauvageot N, Gilson G, Stranges S (2015) Prevalence and correlates of vitamin D deficiency and insufficiency in luxembourg adults: Evidence from the observation of cardiovascular risk factors (ORISCAV-LUX) study. Nutrients 7:6780–6796. https://doi.org/10.3390/nu7085308

Anderson DR (2008) Model Based Inferences in the Life Sciences: A primer on Evidence, 1st edn. Springer Science+Business Media, LLC, New York

Bleizgys A, Kurovskij J (2018) Vitamin D levels of out-patients in Lithuania: Deficiency and hypervitaminosis. Medicina (Lithuania) 54:. https://doi.org/10.3390/medicina54020025

Borissova AM, Shinkov A, Vlahov J, et al (2013) Vitamin D status in Bulgaria - Winter data. Arch Osteoporos 8:4–8. https://doi.org/10.1007/s11657-013-0133-4

Colin Cameron A, Windmeijer FAG (1997) An R-squared measure of goodness of fit for some common nonlinear regression models. J Econom 77:329–342. https://doi.org/https://doi.org/10.1016/S0304-4076(96)01818-0

EBMPHET Consortium (2020) COVID-19 Severity in Europe and the USA: Could the Seasonal Influenza Vaccination Play a Role? [Preprint]. SSRN. https://doi.org/10.2139/ssrn.3621446

Elmadfa I, Meyer A, Wottawa D, et al (2018) Vitamin D Intake and Status in Austria and Its Effects on Some Health Indicators. Austin J Nutr Metab 4:1050

Hale T, Angrist N, Goldszmidt R, et al (2021) A global panel database of pandemic policies (Oxford COVID-19 Government Response Tracker). Nat Hum Behav. https://doi.org/10.1038/s41562-021-01079-8

Hribar M, Hristov H, Gregorič M, et al (2020) Nutrihealth Study: Seasonal Variation in Vitamin D Status among the Slovenian Adult and Elderly Population. Nutrients 12:1838. https://doi.org/10.3390/nu12061838

Ilie PC, Stefanescu S, Smith L (2020) The role of vitamin D in the prevention of coronavirus disease 2019 infection and mortality. Aging Clin Exp Res 32:1195–1198. https://doi.org/10.1007/s40520-020-01570-8

Lips P, Cashman KD, Lamberg-Allardt C, et al (2019) Current Vitamin D status in European and Middle East countries and strategies to prevent Vitamin D deficiency: A position statement of the European Calcified Tissue Society. Eur J Endocrinol 180:P23–P54. https://doi.org/10.1530/EJE-18-0736

Panarese A, Shahini E (2020) Letter: Covid-19, and vitamin D. Aliment Pharmacol Ther 51:993–995. https://doi.org/10.1111/apt.15752

Rabenberg M, Mensink GBM (2016) Vitamin-D-Status in Deutschland. J Heal Monit 1:36–42. https://doi.org/10.17886/RKI-GBE-2016-036

Rumano M, Mitre A, Rumano E (2019) Vitamin D Status And Parathyroid Hormone In Albanians. In: The Eurasia Proceedings of Educational & Social Sciences. Cesme, Turkey, pp 106–110

Santos A, Amaral TF, Guerra RS, et al (2017) Vitamin D status and associated factors among Portuguese older adults: Results from the Nutrition UP 65 cross-sectional study. BMJ Open 7:1–13. https://doi.org/10.1136/bmjopen-2017-016123

Sokolovic S, Alimanovic-Alagic R, Dzananovic L, et al (2017) Vitamin D status in Bosnia and Herzegovina: the cross-sectional epidemiological analysis. Osteoporos Int 28:1021–1025. https://doi.org/10.1007/s00198-016-3831-0

Spiro A, Buttriss JL (2014) Vitamin D: An overview of vitamin D status and intake in Europe. Nutr Bull 39:322–350. https://doi.org/10.1111/nbu.12108

Sterne JAC, White IR, Carlin JB, et al (2009) Multiple imputation for missing data in epidemiological and clinical research: potential and pitfalls. BMJ Br Med J 338:b2393. https://doi.org/10.1136/bmj.b2393

Van Buuren S, Groothuis-Oudshoorn K (2011) mice: Multivariate Imputation by Chained Equations in R. J Stat Softw 45:1–67. https://doi.org/10.1177/0962280206074463

1. Albania, Andorra, Austria, Belarus, Belgium, Bosnia and Herzegovina, Bulgaria, Croatia, Czechia, Denmark, Estonia, Finland, France, Germany, Greece, Hungary, Iceland, Ireland, Isle of Man, Italy, Latvia, Liechtenstein, Lithuania, Luxembourg, Malta, Moldova, Monaco, Montenegro, Netherlands, North Macedonia, Norway, Poland, Portugal, Romania, Russia, San Marino, Serbia, Slovakia, Slovenia, Spain, Sweden, Switzerland, UK, Ukraine; the “Europe” tab of Worldometers’ Covid-19 site does not contain data for Cyprus [↑](#footnote-ref-1)
